# Therapeutic Potential of *Punica granatum* and Isolated Compounds: Evidence-Based Advances to Treat Bacterial Infections

**DOI:** 10.1155/2023/4026440

**Published:** 2023-12-15

**Authors:** Priscila Mendonça Mendes, Guilherme Martins Gomes Fontoura, Liliane dos Santos Rodrigues, Aloiso Sampaio Souza, Jesse Pereira Machado Viana, Ana Lucia Fernandes Pereira, Richard Pereira Dutra, Adriana Gomes Nogueira Ferreira, Marcelino Santos Neto, Aramys Silva Reis, Andresa Aparecida Berretta, Valério Monteiro-Neto, Márcia Cristina Gonçalves Maciel

**Affiliations:** ^1^Department of Biology, Federal University of Maranhão, São Luís 65080-805, Brazil; ^2^Graduate Program in Health and Technology, Center of Sciences in Imperatriz, Federal University of Maranhão, Imperatriz 65915-240, Brazil; ^3^Department of Medicine, Afya Faculty of Medical Sciences, Bragança 68600-000, Brazil; ^4^Department of Cell Biology, University of Brasília, Brasília 70910-900, Brazil; ^5^Research, Development & Innovation Department, Apis Flora Industrial e Comercial Ltda, São Paulo 14020-670, Brazil; ^6^Department of Pathology, Federal University of Maranhão, São Luís 65080-805, Brazil

## Abstract

*Punica granatum* Linn has been known for its nutritional and medicinal value since ancient times and is used in the treatment of various pathologies owing to its antibacterial properties. This review reports the results of the most recent studies on the antibacterial effects of *P. granatum* and its isolated compounds on bacteria of clinical interest. A search in the PubMed, Scopus, Science Direct, and Science Citation Index Expanded (Web of Science) databases was performed, which included articles that evaluated the antibacterial activity of *P. granatum* extracts and excluded articles that analyzed other microorganisms or nonpathogenic bacteria, as well as theses, dissertations, duplicate articles, and those not fully available. The literature suggests that *P. granatum* extracts can act on bacteria, such as methicillin-sensitive *Staphylococcus aureus* (MSSA), methicillin-resistant *S. aureus* (MRSA), *Streptococcus mutans*, *Escherichia coli*, *Pseudomonas aeruginosa*, and *Klebsiella pneumoniae*. In addition, fruit peel was the most commonly used pharmacogen and methanol, ethanol, and water were the most common solvents for the extraction of bioactive compounds. The antibacterial potential of the methanolic extract of pomegranate peel could be attributed to the presence of active compounds, such as 5-hydroxymethylfurfural, punicic acid, gallic acid, and punicalagin. Thus, there is evidence that these plant extracts, having high polyphenol content, can disrupt the bacterial plasma membrane and inhibit the action of proteins related to antimicrobial resistance. *P. granatum* shows antibacterial activity against Gram-positive and Gram-negative bacteria, with great potential against multidrug-resistant strains. Further research is needed to clarify the mechanism of action related to this biological activity and investigate the isolated substances that may be responsible for the antibacterial effects.

## 1. Introduction

The multidrug-resistant (MDR) bacteria are creating a serious challenge to treat diseases, and the essential oils from medicinal plants are effective natural products for resistant pathogen bacteria [1–4]. Medicinal plants are widely used as herbal medicines, and the World Health Organization (WHO) recognizes that much of the population of developing countries depends on traditional medicine for their primary care; 80% of this population uses traditional practices in their basic health care and 85% uses plants or their preparations. Since the Declaration of Alma Ata in 1978, in which the use of medicinal plants and herbal medicines for prophylactic, curative, and palliative purposes was recognized, the WHO has encouraged the use of medicinal plants for healthcare [5].

Natural products have proven to be alternative and potential sources of synthetic drugs. Studies have shown that crude extracts or purified chemical constituents of various medicinal plants are often more effective than synthetic antioxidants [6]. *P. granatum* (pomegranate) has been widely used in the food and pharmaceutical industries for a variety of applications and as a source of antioxidants to combat autoxidation-induced pathologies [7, 8]. *Punica granatum* Linn, belonging to the family Punicaceae, is well known for its nutritional and medicinal value [9]. Approximately, 50% of the total weight of fruits corresponds to the peel, which is an important source of bioactive compounds, such as phenolics, flavonoids, ellagitannins, and minerals, which primarily include potassium, nitrogen, calcium, phosphorus, magnesium, sodium, and complex polysaccharides [10].

In addition to its nutritional benefits, the fruit is used empirically for the treatment of various diseases, such as acidosis, dysentery, microbial infections, parasitic infections, hemorrhage, and respiratory pathologies [9, 11]. The pharmacological properties of pomegranate have a long history, and recent decades have seen a growing interest in its therapeutic effects [6].

Research has evidenced that pomegranate extracts can be used as natural alternatives for the treatment of various diseases, including bacterial and viral infections [12]. The antimicrobial potential of *P. granatum* extracts can be attributed to its high content of hydrolyzable polyphenols, consisting mainly of gallotannins and ellagitannins, such as punicalagin, punicalins, and ellagic acid [9].

Gram-negative bacteria, such as *Escherichia coli*, *Pseudomonas aeruginosa*, and *Proteus vulgaris*, and Gram-positive pathogens, such as *Staphylococcus aureus* and *S*. *epidermidis*, are responsible for most hospital infections [13–16]. Extracts of *P. granatum* were potent inhibitors of several bacteria of clinical interest, including *Listeria monocytogenes*, *S*. *aureus*, *E*. *coli*, and *Salmonella typhimurium* [17].

As they provide low-cost therapeutic potential, there is considerable interest in the use of plant products as an alternative method to control pathogenic microorganisms and many natural product compounds have been observed to target pathogenic bacteria [7].

There is still much to explore regarding the antibacterial activity of this natural product, for instance, to improve the existing technologies or make them more accessible through the development of simpler, cheaper, and equally efficient technologies. These must utilize raw materials found in less-developed regions, as some of the challenges in treating bacterial infections are the high cost of treatment, bacterial resistance, and inaccessibility of remote locations.

Therefore, this study aimed to conduct a literature review of the antibacterial effects of *P. granatum* and its isolated compounds on bacteria of clinical interest by synthesizing data on the production of extracts, frequently used evaluation methods, and the main results described in these studies. It also provides direction for future investigations to expand the chemical and biological knowledge of this species and highlights its most promising antibacterial compounds that are worthy of further exploration.

## 2. Materials and Methods

In this study, we performed an integrative review of the literature according to the following steps: elaboration of the guiding question, search for primary studies, evaluation of primary studies, data extraction, analysis, and synthesis of results and presentation.

### 2.1. Identifying the Research Question

For the elaboration of the guiding question, the PICO strategy was used [18], in which “P” refers to the study population (bacteria of clinical interest); “I” is the intervention studied or the variable of interest (*P. granatum* used as the antibacterial agent); and “CO” is the context, which refers to the outcome of interest (antibacterial activity). Thus, the guiding question for this integrative review was “does the species *P. granatum* provide effective antibacterial action against bacteria of clinical interest?”

### 2.2. Search Strategy

For the selection of articles in the literature, a search was performed using the following: PubMed, Science Direct, Scopus, and Science Citation Index Expanded (Web of Science); accessed: February 15^th^–March 5^th^, 2022, by the CAPES scientific journals gateway. The following controlled descriptors, keywords, and synonyms with Boolean operators were used for crossing the database: “Antibacterial Agents” OR “Agents, Antibacterial” OR “Antibacterial activity” AND “Pomegranate” OR “Pomegranates” OR “*Punica granatum*.” The data were collected between February and March 2022.

### 2.3. Inclusion and Exclusion Criteria

Articles that evaluated the antibacterial activity of *P. granatum* extracts without language restrictions or time periods were included. Articles that analyzed other microorganisms or nonpathogenic bacteria and those that associated *P. granatum* with extracts of other natural products or antibiotics were excluded. In addition, theses, dissertations, duplicate articles, and articles that were not fully available were excluded.

### 2.4. Summarizing and Reporting the Results

The selected articles were read in full for critical analysis and categorization. According to the adopted design, all were found to be *in vitro* preclinical trials [19].

These studies were also reviewed to extract data, such as the pharmacogen used, solvent extractor, bacterial strain, and antimicrobial susceptibility assays. The results were analyzed to identify whether there was inhibition of bacterial growth expressed by inhibition zones (mm) and to determine the minimum inhibitory concentration (MIC) and/or the minimum bactericidal concentration (MBC). Subsequently, the major compounds that may be responsible for the biological activity of the species were verified. Subsequently, their molecular structures were illustrated with the help of ChemSketch software (ACD/Labs, version 2021.2.1).

## 3. Results and Discussion

Medicinal plants are widely recognized as safe treatment options, providing protective effects against a broad spectrum of toxic agents, whether natural or synthetic [20, 21]. These agents not only neutralize the negative effects of toxicants [22] but also play a crucial role in adjusting immunity-related genes, thereby boosting the body's defense against infections and infestations [23, 24] Among these, *P. granatum* has garnered extensive attention due to its constituents' involvement in various biological and pharmacological activities, contributing to overall health benefits [25]. In this review article, we have focused on the direct action and immunomodulatory effects of pomegranate against bacterial infections.

**Figure 1:**
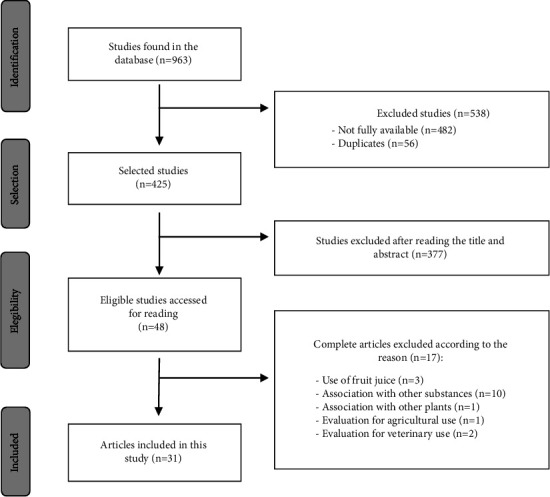
Flowchart for study selection according to PRISMA.

In search of this information, articles were selected, including 963 primary studies. Of these, 56 were excluded owing to duplication, 482 were not fully available, 377 were excluded after reading the title and abstract, and 17 did not answer the guiding question. The final analysis included 31 articles that were published between 2002 and 2020. The selection of the studies is presented in a flowchart (Figure 1), as recommended by the PRISMA group [26].

The majority of the articles were written in English (*n* = 30, 93.5%), with only one in Portuguese (*n* = 1, 6.5%). There was a higher number of articles published between the years 2011 and 2020 (*n* = 26, 83.9%), while fewer relevant articles were published during 2002–2010 (*n* = 5, 16.1%); thus, there was a progressive increase in studies on the antibacterial activity of *P. granatum* (Table 1). Most studies were conducted in Asia (*n* = 21, 67.6%), mainly in India (*n* = 6, 16.1%) and Iran (*n* = 4, 23.9%). The origins of pomegranate are believed to be in the Asian Mediterranean, particularly in parts of Iran and northern India, where it spread to the rest of the world. Hence, research is commonly concentrated in this region because of its long history of pomegranate use in healthcare [38].

The main bacterial strains used in the studies were methicillin-sensitive *S. aureus* and methicillin-resistant *S. aureus* (*n* = 16, 51.6%), and *Streptococcus mutans* (*n* = 11, 35.5%) as Gram-positive bacteria; *E*. *coli* (*n* = 16, 51.6%) and *Klebsiella pneumoniae* (*n* = 9, 29.0%) as Gram-negative bacteria; and *Mycobacterium smegmatis*, *Mycobacterium bovis*, and *Mycobacterium tuberculosis* (*n* = 1, 3.2%) as mycobacteria. The reasoning for investigating the antibacterial activity of *P. granatum* against these strains may be related to the frequency with which they are involved in infectious diseases acquired in the community and hospital environment, as some of these species have a high capacity to develop antimicrobial resistance [52]. Studies investigating the antibacterial activity of natural products found that traditional medicine is facing great challenges in overcoming antimicrobial resistance to commercial antibiotics; therefore, one of its main goals is to find alternative compounds capable of combating or reducing bacterial resistance.

The commonly used methods for evaluating the antibacterial activity of natural extracts were used in the studies, such as the MIC (*n* = 22, 71.0%), MBC (*n* = 12, 38.7%), and inhibition halo (*n* = 23, 74.2%). These assays have been used to determine the antimicrobial activity of extracts of *P. granatum* and several other plant products. The broth dilution assay and the agar diffusion method are more commonly used because of their low cost, speed, and ease of performance compared to other tests [53].

The extracts obtained from the plant *in natura* were specifically used as pharmacogens, including the peel (*n* = 21, 67.7%), leaves (*n* = 5, 16.1%), fruit/pulp (*n* = 4, 12.9%), flowers (*n* = 4, 12.9%), water (*n* = 12, 38.7%), ethyl acetate (*n* = 4, 12.9%), hydroalcohol (*n* = 4, 12.9%), hexane (*n* = 3, 9.7%), and chloroform (*n* = 3, 9.7%). The choice of fruit peel as the main pharmaceutical for medicinal use can be justified by the amount of phenolic compounds found in this portion, which is generally discarded as waste in the food industry, making it an ideal pharmaceutical for medicinal use [54].

**Figure 2:**
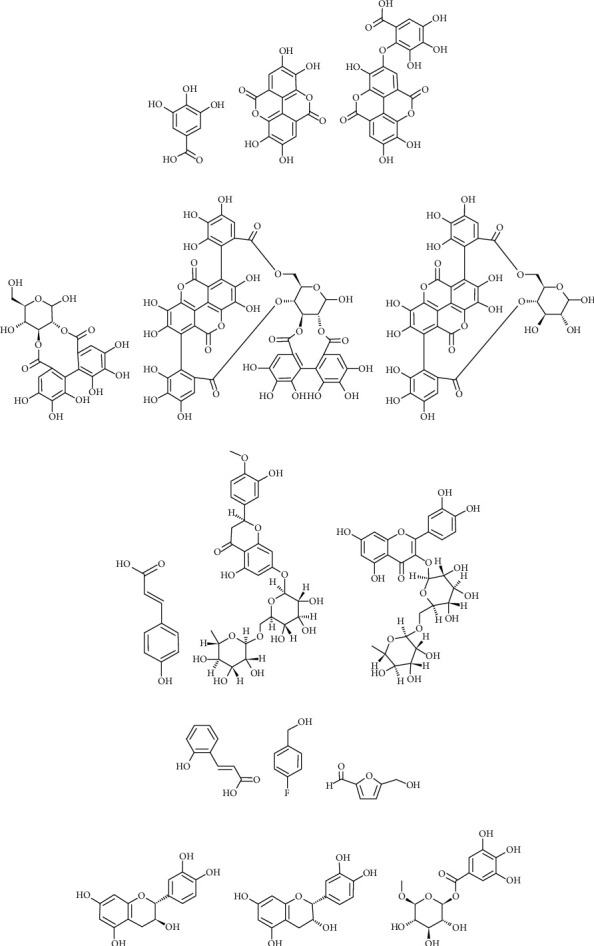
Structures of compounds identified in *P. granatum* extracts. (a) Gallic acid, (b) ellagic acid, (c) valoneic acid dilactone, (d) hexahydroxydiphenoyl-hexoside, (e) punicalagin, (f) punicalin, (g) p-coumaric acid, (h) hesperidin, (i) rutin, (j) coumaric acid, (k) 4-fluorobenzyl alcohol, (l) 5-hydroxymethylfurfural, (m) catechin, (n) epicatechin, and (o) monogalloyl-hexoside.

**Figure 3:**
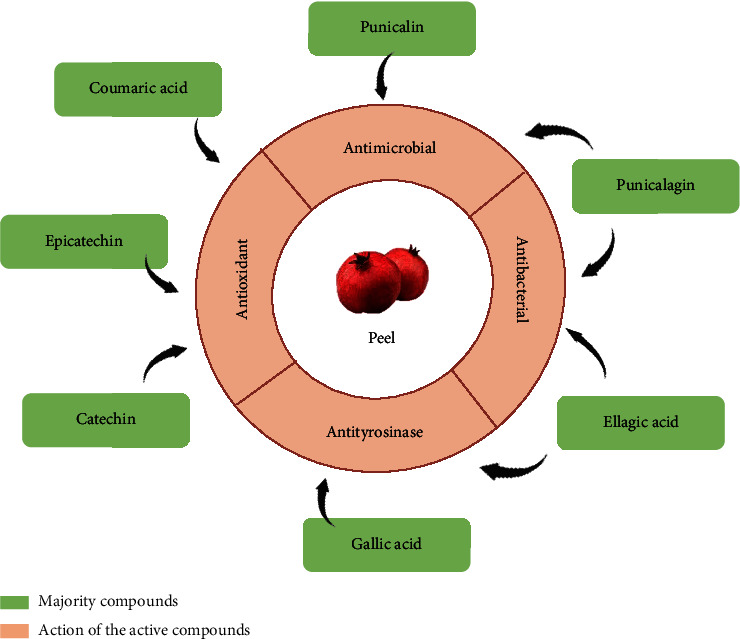
Effects of the *P. granatum* bark extract and its main bioactive compounds.

Regarding the solvent extractor, the selectivity, solubility, cost, and safety should be considered when choosing the optimal solvent. Solvents with a polarity value close to that of the solute exhibit better performance, with alcohols (EtOH and MeOH) being the universal solvents for extraction in phytochemical investigations [55]. The main classes of the identified phytochemical components (Table 2 and Figures 2 and 3) of *P. granatum* are phenolic acids (ellagic and gallic acids), flavonoids (quercetin and anthocyanins), and hydrolyzable tannins (punicalin and punicalagin) [57], all of which have higher solubility in polar solvents, which explains the choice of using MeOH and EtOH as the main extraction agents.

As traditional drug therapies are gradually losing their effectiveness, new therapies based on natural antimicrobial compounds are emerging as alternative treatments for hospital infections [58]. Natural products, such as extracts of the *P. granatum* fruit and other parts containing a wide range of biomolecules, are commonly used because of their antimicrobial activity. The antimicrobial properties of plant extracts may differ because of variations in their mode of action and chemical composition. Many factors can influence the antimicrobial activity, such as plant freshness, pharmacogen, extraction method, solvent, geographic region where the plant is grown, and growing season [59]. In this study, the most used plant pharmacogens were the bark (>50% of the studies) and leaves, followed by extracts of the flowers, fruit, petal, stem, pulp, or peel/pulp. Of these, only one did not indicate the portion used to prepare the extract.

Previous studies have shown that compared to other parts of the pomegranate, the peel contains a higher concentration of phenolic compounds, such as flavonoids, phenolic acids, and hydrolyzable tannins as the main compounds [60]. These compounds vary in their chemical nature and play significant roles in the plant's antimicrobial, anti-inflammatory, and antioxidant activities [61]. Thus, the phenolic content of the pomegranate peel is up to three times greater than that in the pulp, which is discarded as biowaste [62]. Owing to this high phenolic content, the food industry has been exploring these residues as sources of natural ingredients with possible applications in new products [63].

Pomegranate possesses pharmacological properties, including antiviral and antimicrobial activities conferred by the high polyphenol concentrations, an effect directly attributed to the presence of bioactive compounds of hydrolyzable tannins [64]. The tannin synthesis pathway is regulated by transcription factors expressed during fruit development. Studies have indicated increased expression of the shikimate dehydrogenase and UDP-glucosyltransferase genes of the shikimate pathway, suggesting increased expression of tannins and differential expression at rind maturation compared to earlier developmental stages of the fruit [65].

Pomegranate peel is an important source of natural bioactive compounds, such as gallic acid, ellagic acid, punicalagin, catechin, and epicatechin, which are also considered the main bioactive compounds in pomegranate bark [66, 67]. Several researchers have reported the biological activities (antimicrobial, antioxidant, anticancer, antimutagenic, and anti-inflammatory) and functions of pomegranate peel. However, some significant factors can interfere with the nutritional quality and phytochemical activities of this species, such as the drying methods used to remove water and reduce chemical reactions. The main drying methods include sun drying, vacuum drying, freeze drying, and oven drying. Furthermore, lyophilization is a potential method for the extraction and recovery of bioactive compounds and other phytochemicals from natural plant sources; however, despite being the best at maintaining the product quality, it is still expensive compared with other methods [68].

Nevertheless, the extraction of bioactive compounds from plant sources is a significant step, and different solvent systems (polar and nonpolar) have been used to extract and recover polyphenols from plant materials. Generally, nonpolar and low-polarity solvents are used to extract lipophilic compounds and pigments from plants [69]. However, the recovery of these substances depends on the type of solvent used and the extraction procedure, which seek to avoid chemical modification of the phenolic compounds. Water, ethanol, methanol, and acetone have been described as the most commonly used solvents [70]. However, our review revealed that the solvents most frequently used for extracting bioactive compounds were methanol, ethanol and chloroform, hexane, hydroalcohol, and butanol.

Recent studies have indicated that extracts of the bark, leaves, stem, pulp, and whole fruit of pomegranate have effective antimicrobial activity, including against multiresistant strains [29, 30, 71]. Moreover, they demonstrated the antibacterial activity of pomegranate against bacteria commonly involved in upper gastrointestinal tract infections. Bark extracts could inhibit the growth of *Streptococcus mutans* (MIC: 10 *µ*g/*µ*L and MBC: 25 *µ*g/*µ*L) and clinical isolates of *Rothia dentocariosa* [27]. Another study with similar results showed inhibition of *Streptococcus mutans* (MIC: 10–25 *µ*g/*µ*L and MBC: 15–40 *µ*g/*µ*L) and *R*. *dentocariosa* (MIC: 10–20 *µ*g/*µ*L and MBC: 15–140 *µ*g/*µ*L) [8].

*In vitro* microbiological tests demonstrated that the peel, pulp, and petal extracts of pomegranate inhibited the growth of the main cariogenic bacteria involved in dental caries. Although these are preliminary data, the authors suggested that pomegranate polyphenolic compounds may represent good adjuvants for the prevention and treatment of dental caries but clinical studies are needed to further evaluate their potential [35, 41, 43, 47, 49].

Pomegranate does not exhibit antibacterial activity only against bacteria that cause oral infections, as the literature has also shown evidence of inhibitory activity against pathogens that cause intestinal infections. Bacteria known to be the main causes of food infections were tested using the agar diffusion assay. The *P. granatum* leaf extract inhibited bacterial growth (9.6 mm) and showed the highest concentration index (ΣFIC) when combined with tetracycline (ΣFIC = 0.37) against a strain of *E. coli* producing metallo-*β*-lactamase 1 (NDM-1) [31], whereas the peel extracts inhibited the growth of resistant clinical isolates of *E. coli* (12–13 mm) [11].

Among urinary tract infections (UTIs) agents, *E. coli* is considered the most frequently isolated and common uropathogen of community- and hospital-acquired infections in 70–90% of UTIs [72]. An analysis of the inhibitory effect of three products, namely, crude pomegranate rind extract (PGRE), punicalagin (PG), and pomegranate powder (PGP), on flagellin gene expression and motility of *E. coli* uropathogenic strain (UPEC CFT073) was performed. The results showed that normalized luminescence decreased with increasing concentrations of PGRE, PG, or PGP, whereas a decrease in *fliC* gene transcription correlated with a decrease in the luminescent signal, suggesting that growth in the presence of the products resulted in reduced *fliC* expression. Thus, this study revealed that PG, PGP, and PGRE at 10% concentration reduced the normalized luminescence signal to 12%, 30%, and 8% of the control signal, respectively, with the strongest inhibitor of *fliC* expression being PGRE at 10% concentration, resulting in impaired bacterial motility and reduction in the spread of infection to the upper urinary tract [73].

*P. granatum* also demonstrated bacteriostatic and bactericidal activities against foodborne strains of pathogenic bacteria, inhibiting the growth of *E. coli* (14.2 mm), *S. aureus* (18.5 mm), *Bacillus cereus* (16.3 mm), *Salmonella typhi* (9.7 mm), and *P. aeruginosa* (16.1 mm) [33]. Similar results were found regarding the antibacterial activity of the *P. granatum* bark extract on a strain of *E. coli* (15–22 mm) as well as other foodborne bacteria, such as *Salmonella typhimurium* (15–23 mm), *Salmonella infantis* (13–18 mm), *Salmonella enterica* (15–28 mm), and *Salmonella brunei* (13–23 mm) [43]. Several studies have evidenced the effects of *P. granatum* extracts against different bacteria. Moreover, among the bacterial species described in the literature, some are resistant to conventional antibiotics.

The main bacteria capable of developing antibacterial resistance include MRSA and *P. aeruginosa*. MRSA is a multidrug-resistant bacterium that causes nosocomial and community-acquired infections worldwide, usually causing severe infectious diseases, including pyogenic endocarditis, osteomyelitis, and pyogenic infections of the skin and soft tissues. It has several mechanisms (capsular polysaccharides, surface-associated proteins, toxins, and extracellular enzymes) that increase its virulence [74]. Analysis of the antimicrobial potential of methanolic and ethanolic extracts of the bark of three fruits (pomegranate, orange, and banana) against pathogenic strains revealed that the ethanolic extract had higher antimicrobial activity against MRSA and *S. aureus* [75]. The antibacterial potential of the methanolic extract of pomegranate peel can be attributed to the presence of active compounds, such as 5-hydroxymethylfurfural, punicic acid, gallic acid, and punicalagin [76]. Thus, there is evidence that plant extracts having high polyphenol content can break the bacterial plasma membrane and inhibit the action of proteins related to antimicrobial resistance [77].

In another study using ethanolic extracts of pomegranate peel (PPEs), mass spectrometry revealed that the most prevalent compounds were punicalagin, ellagic acid, granatin, and punicalin. Analysis of the antibacterial activity revealed that *S. aureus* ATCC 25923 showed higher sensitivity with MIC and MBC ranging between 0.8 and 6.4 mg/mL, while another Gram-positive bacterium, *Listeria monocytogenes* ATCC 19115, showed lower sensitivity with MIC and MBC of 12.8 mg/mL [78]. The antibacterial capacity of pomegranate peel extracts depended on the solvent used, resulting in variations in the biological effects or differences in the results, which highlights the importance of selecting an appropriate solvent and extraction method for isolating bioactive compounds.

Infections caused by the opportunistic Gram-negative bacillus *P. aeruginosa* are mainly owed to biofilm formation, which increases its resistance to conventional antibiotics [79]. The use of isolated plant extracts or their combinations has become a promising alternative for inhibiting bacterial biofilms [80]. The susceptibility of *P. aeruginosa* isolates to plant extracts (pomegranate, thyme, cinnamon, rosemary, clove, and peppermint) was investigated using the agar diffusion method, with the results indicating that the extracts exhibited variations in inhibition hanos (12–26 mm). The pomegranate extract showed the largest inhibition zone (26 mm) against *P. aeruginosa*, followed by thyme (20 mm), rosemary (18 mm), cloves (17 mm), ginger (16 mm), and peppermint (12 mm). The data from the present study revealed that pomegranate and rosemary extracts, when tested alone, were able (91.93 and 90.83%, respectively) to inhibit biofilm formation by adhering bacteria to surfaces and reducing colony units and extrapolymer layers. These differences between the extracts can be attributed to the types of active compounds in them. In addition, the combination of both plant extracts with the antibiotic ceftazidime was more effective in eradicating the *P. aeruginosa* biofilm, ranging from 97.3 to 99.6% [81]. Hacioglu et al. [82] reported that pomegranate flower tea in combination with ampicillin had a synergistic effect (FIC = 0.5) against the clinical isolate *E. faecalis*.

The growing importance of natural products and their eventual valorization in traditional medicine are due to the need for new alternatives to combat antimicrobial resistance, which directly interferes with the treatment and healing process of infectious diseases that can lead to more severe conditions, thus increasing the mortality rate. The mechanism of action of *P. granatum* in clinical isolates should be investigated to clarify the processes involved in its antibacterial activity, thereby enhancing the safety and efficacy of their use in healthcare. Our findings highlight the potential use of *P. granatum* extracts in the pharmaceutical industry to develop antimicrobials.

## 4. Conclusions

In conclusion, this review revealed that research on *P. granatum* has been gaining prominence in recent years because it is considered a useful and natural alternative for the development of antibacterial agents. Although the mechanism by which *P. granatum* extracts modulate bacterial resistance remains unclear, the results suggest that they can act on Gram-positive and Gram-negative bacteria with great potential against multidrug-resistant strains. In addition, pomegranate bark was the most commonly used pharmacogen, whereas methanol, ethanol, and water were the most frequently used solvents for the extraction of bioactive compounds. The antimicrobial activity of the methanolic extracts of pomegranate peel could be attributed to the presence of active compounds such as 5-hydroxymethylfurfural, punicic acid, gallic acid, and punicalagin. Moreover, pomegranate extracts with a high polyphenol content were capable of disrupting bacterial plasma membranes and inhibiting the activity of proteins associated with antimicrobial resistance. Further studies should be conducted to clarify the mechanism of action behind this biological activity, including the investigation of isolated substances that may be responsible for the antibacterial potential. As clinical studies are still limited, further research is needed to ensure the safety and quality of therapeutic applications of this natural product.

## Figures and Tables

**Table 1:** Description of materials, methods, and results of studies that evaluated the antibacterial activity of *P. granatum* extracts.

Pharmacogen	Solvent	Bacteria (strain)	Halo inhibition (mm)	MIC	MBC	Reference
Peel	Hydroalcoholic	*S. mutans* ATCC 25175	11.2–16.2	10^b^	25^b^	[27]
		*R. dentocariosa* Rd1 (CI)	12.8–15.7	10	15	

Leaf	MeOH	*S. mutans* UA159	Resistant	—	—	[28]
Stem		*S. sanguinis* DSS-10 ATCC 10 556	Resistant			
Fruit		*E. coli* DH10B	Resistant			

Peel	MeOH	*M. smegmatis*	—	0.1^a^	0.3^a^	[29]
		*M. bovis* BCG		0.2	0.3	

Leaf	—	*S. mutans* DMST18777	16.72	—	250^a^	[30]
		*S. sanguinis* DMST18782	24.50		125	
		*L. casei* BCC36987	Resistant		500	

Fruit	MeOH	*S. aureus* ATCC 6538	12.5	8^a^	8^a^	[17]
		*B. subtilis* ATCC 6633	28.6	4	4	
		*B. cereus* ATCC 11774	28.6	4	6	
		*L. monocytogenes* ATCC 19118	3.14	>10	>10	
		*S. typhimurium* ATCC 14028	3.14	>10	>10	
		*E. coli* O157: H7 ATCC 10536	3.14	>10	>10	

Peel Pulp	Hydroalcoholic	*S. mutans* ATCC 25175	—	10–25^b^	15–40^b^	[12]
		Rd1 *R. dentocariosa* (CI)		10–20	15–140	

Leaf	EtOH	*E. coli* NDM-1 (CI)	9.6	5.12^a^	15–25^a^	[31]

Peel	EtOH MeOH	*P. aeruginosa* (nine CI)	6.66–40.66	—	—	[32]
	H_2_O					

Flower	H_2_O	*P. aeruginosa* ATCC 27853	—	1.25^d^	—	[28]
		*A. baumannii* ATCC 19606		2.50		
		*E. coli* ATCC 25922		1.25		
		*K. pneumoniae* ATCC 4352		1.25		
		*E. faecalis* ATCC 29212		1.25		
		MSSA ATCC 29213		2.50		
		MRSA ATCC 43300		2.50		
		CI similar to standard ATCC used		0.62–2.5		

Peel	H_2_O	*A. baumannii* (CI)	14-15	—	—	[11]
	CHCl_3_	*E. coli* (CI)	12-13			
	EtOH	*P. aeruginosa* (CI)	10–14			
	Hex	*S. aureus* (CI)	11			

Peel	EtOH	*S. aureus*	18.5	2.5^a^	5.0^a^	[33]
		*B. cereus*	16.3	—	—	
		*E. coli*	14.2	—	—	
		*S. typhi*	9.7	—	—	
		*P. aeruginosa*	16.1	2.5	5.0	

Peel	Hex AcOEt MeOH	*E. coli* MTCC 441	8–16	—	—	[34]
		*K. pneumoniae* ATCC 1705	6–9			
		*S. diastaticus* MTCC 1394	13–21			
		*E. faecalis* MTCC 439	6–13			
		*E. aerogenes* (CI)	7–19			
		*K. pneumoniae* (CI)	6–15			
		*E. faecalis* (CI)	7–16			
		*S. epidermidis* (CI)	10			
		*M. smegmatis* (CI)	10–19			
		*E. coli* (CI)	10			

Peel	H_2_O	*S. mutans* ATCC 25175	—	0.02^d^	—	[35]

Peel	MeOH	*E. coli* ATCC 11775	—	0.20–0.39^a^	—	[36]
		*K. pneumoniae* ATCC 13883		0.20–0.39		
		*B. subtilis* ATCC 6051		0.10–0.39		
		*S. aureus* ATCC 12600		0.10–0.39		

Leaf	H_2_O MeOH	*S. aureus*	12	625^b^	—	[37]
		*E. coli*	10	—		
		*B. cereus*	10–17	1250		
		*S. typhimurium*	10–12	—		
		*S. dysenteriae*	—	—		
		*S. flexneri*	10–12	2500		
		*S. sonnei*	—	—		
		*V. cholerae*	12–14	2500		

Flower	Hydroalcoholic	*S. mutans* ATCC 35668, PTCC 1683	9.5–17.5	3.90^a^	3.9^a^	[38]
		*S. sanguinis* ATCC 10556, PTCC 1449	14.5–21.5	7.81	31.25	
		*S. salivarius* ATCC 9222, PTCC 1448	12–16	31.25	62.5	
		*S. sobrinus* ATCC 27607, PTCC 1601	12–22.5	7.81	31.25	
		*E. faecalis* ATCC 11700, PTCC 1393	10.5–15.5	125	250	

Flower	H_2_O EtOH MeOH CHCl_3_ AcOEt	*S. aureus* ATCC 25923	13–34	0.19–3.12^a^	0.78–12.5^a^	[39]
		*B. cereus* PTCC 1247	10–29	0.19–3.12	1.56–12.5	
		*L. monocytogenes* ATCC 7644	10–32	1.56–6.25	6.25–25	
		*E. coli* ATCC 25922	10–28	3.12–12.5	12.5–50	
		*S. dysantriae* PTCC 1188	12–32	0.39–6.25	1.56–25	
		*S. typhi* ATCC 19430	10–31	1.56–6.25	6.25–25	

Leaf	EtOH	*S. aureus* ATCC 25923	12	500^c^	—	[40]
		*S. epidermidis* ATCC 14990	13	31		
		*P. aeruginosa* ATCC 27853	11	—		
		*E. coli* ATCC 14942	10	—		

Peel Pulp	H_2_O MeOH	*M. tuberculosis* (ATCC 25177)	—	128–256^c^	128–512^c^	[9]
		*M. tuberculosis* (CI)		64–>1024	128 ≥ 1204	
		*K. pneumoniae* ATCC 700603		512–>1024	1024 ≥ 1024	
		*K. pneumoniae* ESBL (CI)		512–>1024	512 ≥ 1024	
		*K. pneumoniae* ATCC BAA 1705		256–512	512 ≥ 1024	
		*K. pneumoniae* KPC (CI)		256–>1024	512 ≥ 1024	

Peel	H_2_O	*P. intermedia*	—	—	—	[41]
		*P. gingivalis*	—			
		*S. mutans*	20–34			
		*S. mitis*	25–30			

Peel	EtOH	*S. aureus* ATCC 29213	9.3–28.3	—	—	[42]
		*E. coli* ATCC 10536	10–27.3			
		*M. luteus* ATCC 10240	8–21.3			
		*B. subtilis* ATCC: 6633	11.3–28			
		*P. aeruginosa* (CI)	—			
		*K. pneumoniae* (CI)	9–20.7			

Peel	EtOH	*S. aureus* MTCC 96	16–23	—	—	[43]
		*S. epidermidis* MTCC 435	23–28	512^c^		
		*S. mutans* MTCC 890	13–21	1024		
		*E. coli* MTCC 739	15–22	2048		
		*K. pneumoniae* MTCC 432	14–19	2048		
		*E. aerogenes* MTCC 111	13–21	2048		
		*P. vulgaris* MTCC 742	15–21	2048		
		*P. mirabilis* MTCC 425	15–21	2048		
		*S. typhi* MTCC 733	13–21	2048		
		*S. paratyphi* MTCC 735	15–23	1024		
		*S. typhimurium* MTCC 98	15–23	1024		
		*S. infantis* MTCC 1167	13–18	2048		
		*S. enterica* MTCC 660	15–28	1024		
		*S. brunei* MTCC 1168	13–23	1024		
		*P. aeruginosa* MTCC 424	13–22	1024		
		*B. cepacia* MTCC 1617	15–23	1024		
		*V. parahaemolyticus* MTCC 451	11–14	2048		
		*H. parahaemolyticus* MTCC 1776	13–21	2048		
		*Y. enterocolitica* MTCC 80	25–28	512		

Peel	EtOH	*A. baumannii* ATCC 19606	—	250^c^	—	[44]

Peel	MeOH H_2_O	*E. coli* ATCC 11775	—	0.39–>12.5^a^	—	[45]
		*K. pneumoniae* ATCC 13883		0.20–>12.5		
		*B. subtilis* ATCC 6051		0.20–>12.5		
		*S. aureus* ATCC 12600		0.26–>12.5		

Peel	MeOH	*S. aureus* ATCC 25923	10–22	8^a^	—	[46]
		*B. megaterium* ATCC 14591	14–26	1		
		*E. coli* ATCC 25922	10–23	64		
		*P. aeruginosa* ATCC 27853	10–27	32		
		*K. pneumoniae* ATCC 700603	11–32	16		

Peel	EtOH	*S. typhi* ATCC 19943	13.3–34.2	250^c^	—	[7]
		*S. paratyphi*	14.3–28.5	62.5		

Peel	MeOH	*S. mutans* PTCC 1683	6–9.7	—	—	[47]
		*S. sanguinis* PTCC 1449	6.5–11.5			
		*S. salivarius* PTCC 1448	6.5–9.5			
		*S. aureus* ATCC 25923	7.5–12.5			
		*S. epidermidis* PTCC 1114	11.5–13.5			
		*A. viscosus* PTCC 1202	6–6.5			
		*L. acidophilus* PTCC 1643	6.5–10			

Peel	AcOEt MeOH H_2_O	*L. monocytogenes* ATCC 7644	20	0.5^a^	—	[48]
		*S. aureus* ATCC 6538	13	2		
		MRSA	16	2		
		*B. subtilis* ATCC 6633	15–17	0.5		
		*E. coli* ATCC 10536	16	1		
		*P. aeruginosa* ATCC 9027	18	—		
		*K. pneumoniae* ATCC 10031	16	2		
		*Y. enterocolitica* ATCC 23715	19	0.25		
		*S. enteritidis* ATCC 4931	—	4		

Peel	Hydro-alcoholic	*S. mitis* ATCC 9811	15–25	—	—	[49]
		*S. mutans* ATCC 25175	11–20			
		*S. sanguis* 10557	14–21			
		*S. sobrinus* ATCC27609	12–24			
		*L. casei* ATCC 7469	10–22			

—	H_2_O	*S. aureus* ATCC 25923	17-18	0.2–0.4^a^	3.2–6.3^a^	[50]
	EtOH	MRSA	15.75–18.53	0.2–1.6	1.6–12.5	

Peel	EtOH	*S. aureus* ATCC 29213 MRSA ATCC 33591 MRSA (CI)	20 20 20	61.5^c^ 61.5 61.5	—	[51]
	Hex					
	CHCl_3_ MeOH					
	H_2_O					
	AcOEt					

^a^mg/mL, ^b^*µ*g/*µ*L, ^c^*µ*g/mL, ^d^concentration (%). CI, clinical isolate; MSSA, methicillin-sensitive *S. aureus*; MRSA, methicillin-resistant *S. aureus*; MIC, minimum inhibitory concentration; MBC, minimum bactericidal concentration; AcOEt, ethyl acetate; CHCl_3_, chloroform; EtOH, ethanol; H_2_O, water; Hex, hexane; MeOH, methanol.

**Table 2:** Chemical characterization of *P. granatum* extracts.

Identification techniques	Majority compounds identified	Molecular formula	Action of the active compounds	Reference
HPLC	Punicalagin	C_48_H_28_O_30_	Antibacterial	[30]

HPLC LC-MS/MS	Valoneic acid dilactone Mono galloyl-hexoside Hexahydroxydiphenoyl-hexoside coumaric acid	C_21_H_10_	Antibacterial	[11]
		C_13_H_16_O_10_		
		C_20_H_18_O_14_		
		C_9_H_8_O_3_		

GC-MS	5-Hydroxymethylfurfural	C_6_H_6_O_3_	Antioxidant	[34]
	4-Fluorobenzyl alcohol	C_7_H_7_FO	Antimicrobial	

UPLC LC-MS	Punicalin Rutin p-coumaric acid (+)-Catechin (−)-Epicatechin Hesperidin	C_34_H_22_O_22_	Antioxidant, antibacterial antityrosinase	[36]
		C_27_H_30_O_16_		
		C_9_H_8_O_3_		
		C_15_H_14_O_6_		
		C_15_H_14_O_6_		
		C_28_H_34_O_15_		

HPLC-ESI/MS LC-MS	Gallic acid	C_7_H_6_O_5_	Antibacterial Antioxidant Tyrosinase inhibition	[45]
	Catechin	C_15_H_14_O_6_		
	Epicatechin	C_15_H_14_O_6_		
	Ellagic acid	C_14_H_6_O_8_		
	Rutin	C_27_H_30_O_16_		

HPLC	*α*-Punicalagin	C_48_H_28_O_30_	Antibacterial	[56]
	*β*-Punicalagin	C_48_H_28_O_30_		
	Gallic acid	C_7_H_6_O_5_		
	Ellagic acid	C_14_H_6_O_8_		

HPLC	*α*-Punicalagin	C_48_H_28_O_30_	Antimicrobial	[51]
	*β*-Punicalagin	C_48_H_28_O_30_		
	*α*-Punicalin	C_34_H_22_O_22_		
	*β*-Punicalin	C_34_H_22_O_22_		

GAE, gallic acid; CE, catechin; HPLC, high-performance liquid chromatography; LC-MS/MS, liquid chromatography-tandem-mass spectrometry; GC-MS, gas chromatography-mass spectrometry; UPLC, ultraperformance liquid chromatography; LC-MS, liquid chromatography mass spectrometry.

## Data Availability

The data used to support the findings of this study are included within the article.
